# Comprehensive pan-cancer analysis reveals prognostic significance of *CENPM* and its role in immune infiltration

**DOI:** 10.1016/j.gendis.2025.101815

**Published:** 2025-08-16

**Authors:** Jinyuan Tang, Sihang Zhang, Yongshuai Jiang, Mingming Zhang

**Affiliations:** a The Second Affiliated Hospital, Harbin Medical University, Harbin, Heilongjiang 150001, China; b College of Bioinformatics Science and Technology, Harbin Medical University, Harbin, Heilongjiang 150001, China

Centromere protein M (CENPM) is a critical component of the constitutive centromere-associated network, playing a significant role in the assembly of kinetochores and the organization of chromosomes.^1^ Emerging evidence suggests that centromere protein family members contribute to cancer progression through multifaceted regulatory mechanisms,^2^^,^^3^ with *CENPM* overexpression implicated in tumor advancement through its oncogenic pathways.^4^ However, the biological implications and immune significance of *CENPM* in pan-cancer remain to be further understood. Through integrative multi-omics analysis of 33 cancer types, we revealed that *CENPM* is aberrantly overexpressed in 28 malignancies and serves as a robust prognostic predictor across multiplecancer types. Mechanistically, *CENPM* was observed to fuel genomic instability via synergistic interactions with tumor mutational burden (TMB), microsatellite instability (MSI), and mismatch repair (MMR) pathways, while concurrently shaping immunosuppressive microenvironments through myeloid-derived suppressor cell (MDSC) infiltration. Functional enrichment analyses further implicated *CENPM* in ribosome biogenesis and cell cycle regulation, bridging mitotic dysregulation to tumor progression. Critically, we demonstrate that *CENPM* operates as an immunological “switch” determining MDSC infiltration’s clinical impact. These findings reveal *CENPM* as both a prognostic indicator and a promising target for immunotherapy strategies across diverse malignancies.

To explore the pan-cancer role of *CENPM*, we initially analyzed the expression data of 33 cancer types. Differential expression analysis using Tumor Immune Estimation Resource 2.0 (TIMER2.0) revealed significant *CENPM* mRNA up-regulation in 19 malignancies versus normal tissues (Fig. S1A). Integration of The Cancer Genome Atlas (TCGA) and Genotype-Tissue Expression (GTEx) datasets expanded normal tissue sampling, identifying additional nine up-regulated cancers (Fig. 1A). Clinical Proteomic Tumor Analysis Consortium (CPTAC) proteomic analysis also revealed elevated *CENPM* expression in multiple cancers (Fig. S1B). Consistent results of immunohistochemistry from the Human Protein Atlas (HPA) database supported these findings (Fig. S1C). Multi-platform evidence demonstrated that *CENPM*’s pan-cancer differential expression potentially implicated complex tumorigenic mechanisms.

**Figure 1:**
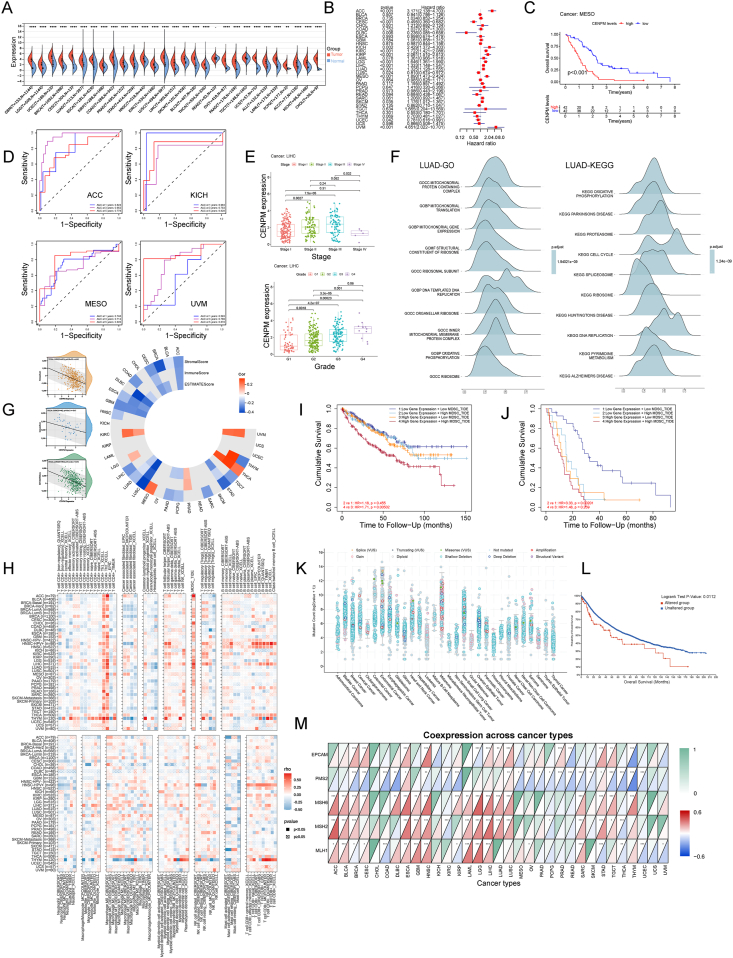
Integrative analysis reveals the role of *CENPM* in pan-cancer. **(A)** The differential expression of *CENPM* mRNA in various tumors and normal tissues from the TCGA and GTEx databases. **(B)** The pan-cancer Cox regression forest plots depicting the correlation between *CENPM* expression and patient overall survival (OS). **(C)** Kaplan–Meier analysis of OS in mesothelioma (MESO). **(D)** Receiver operating characteristic curve of *CENPM* in four types of cancer. **(E)** Correlation analysis between CENMP expression and the stage and grade of liver hepatocellular carcinoma (LIHC). **(F)** Gene Set Enrichment Analysis (GSEA) of *CENPM* in lung adenocarcinoma (LUAD). **(G)** Correlation analysis between *CENPM* expression and stromal score/immune score/ESTIMATE score in 33 tumors, with a Scatter plot of correlation between *CENPM* expression and different scores in representative cancers. **(H)** Immune cell infiltration analysis of *CENPM* in pan-cancer. **(I, J)** Effects of *CENPM* expression and myeloid-derived suppressor cell (MDSC) infiltration on the prognosis of patients with kidney renal clear cell carcinoma (KIRC) (I) and cervical squamous cell carcinoma/endocervical adenocarcinoma (CESC) (J). **(K)** Mutation counts of *CENPM* in TCGA tumors. **(L)** Analysis of the correlation between mutation status and OS in pan-cancer using the cBioPortal tool. **(M)** The correlations between *CENPM* and levels of five genes that are key to intact mismatch repair (MMR) functions in multiple cancers. ∗*p* < 0.05, ∗∗*p* < 0.01, ∗∗∗*p* < 0.001, and ∗∗∗∗*p* < 0.0001.

Univariate Cox regression analysis revealed *CENPM*’s dual prognostic role across malignancies. Elevated *CENPM* predicted worse overall survival in adrenocortical carcinoma, kidney chromophobe, kidney renal clear cell carcinoma, *etc*., yet paradoxically correlated with improved overall survival in cervical squamous cell carcinoma, endocervical adenocarcinoma, diffuse large B-cell lymphoma, lung squamous cell carcinoma, *etc* (Fig. 1B). It showed negative associations with disease-free interval in breast invasive carcinoma, kidney renal papillary cell carcinoma, liver hepatocellular carcinoma, *etc*., while demonstrating divergent impacts on disease-specific survival and progression-free interval depending on cancer type (Fig. S2). Kaplan–Meier validation confirmed its role as a context-dependent pan-cancer prognostic modulator (Fig. 1C; Fig. S3 and S4). Time-dependent receiver operating characteristic curves validated *CENPM*’s excellent predictive capacity for overall survival, particularly in adrenocortical carcinoma, kidney chromophobe, mesothelioma, and uveal melanoma (Fig. 1D). We subsequently investigated the relationship between *CENPM* expression and clinical pathology across multiple cancers, revealing that overexpression of *CENPM* was predictive of pathological stage and histological grade (Fig. 1E; Fig. S5). Gene Set Enrichment Analysis (GSEA) was conducted based on Gene Ontology (GO) and Kyoto Encyclopedia of Genes and Genomes (KEGG). The results showed that expression of *CENPM* was related to “organelle ribosomes”, “ribosomal subunits”, “DNA replication”, “cell cycle”, and other biological processes (Fig. 1F; Fig. S6; Table S1). Another study also demonstrated that *CENPM* regulated the phosphorylation level of ribosomal protein p70S6 kinase via mTOR activation and ultimately promoted pancreatic cancer,^5^ which partially elucidates the aberrant expression of *CENPM* observed in various tumors and its correlation with unfavorable prognoses in patients.

We further investigated the correlations between *CENPM* expression and stromal score/immune score/ESTIMATE score across a range of cancers. These findings demonstrated that *CENPM* expression significantly correlated with the three scores (Fig. 1G; Table S2), suggesting that *CENPM* may exert complex and tumor-specific effects on immune infiltration. The TIMER2.0 platform was used to investigate the correlations between *CENPM* expression and immune cell infiltration. Integrated results from multiple algorithms determined that *CENPM* expression was significantly associated with the infiltration levels of several immune cell types, including CD4^+^ T cells, regulatory T cells, B cells, tumor-associated fibroblasts, monocytes, macrophages, and MDSCs (Fig. 1H). Notably, the infiltration of Th1 and Th2 CD4^+^ T cells exhibited a strong positive correlation with *CENPM* expression across pan-cancer types, while MDSC cells were identified in 31 different cancer types, showing consistent positive correlation. Conversely, the infiltration of mast cells was found to be negatively correlated with *CENPM* expression. It is posited that the expression of *CENPM* may serve as a significant regulatory element affecting the infiltration of these immune cells. The expression of *CENPM* exhibits strong correlation with the expression of various immune cell types in thymoma, suggesting that *CENPM* may play a significant role in shaping the tumor immune microenvironment. Similar outcomes are obtained through the analysis of the TISIDB database (Fig. S7A).

Systematic analyses of the correlations betweenCD4^+^ T cell and MDSC infiltration and clinical outcomes indicate that *CENPM* expression and the infiltration of immune cells have a significant impact on patient prognosis (Fig. S8). Notably, the expression of *CENPM* in certain tumors functions akin to a “switch”, whereby the impact of immune cell infiltration on patient survival is contingent upon whether *CENPM* is expressed at specific low or high levels. In detail, high *CENPM* expression in specific tumors could lead to a negative correlation between MDSC infiltration and survival (Fig. 1I; Fig. S9A–S9F); whereas in other cancers, low *CENPM* levels may result in MDSC infiltration correlating with worsened prognosis (Fig. 1J; Fig. S9G and S9H). Targeted inhibition of *CENPM* may offer a novel therapeutic strategy by directly suppressing malignancies and harnessing its regulatory “switch” function, improving the efficacy of existing treatment modalities. Moreover, the relationship between *CENPM* and several categories of immune-related genes demonstrated that *CENPM* exhibited significant correlations with diverse immune-related genes in most cancer types (Fig. S7B–S7F), suggesting that *CENPM* plays a crucial role in tumor microenvironment-mediated immune responses.

The analysis of genetic alteration revealed that *CENPM*’s mutation spectrum exhibited significant cancer-type specificity (Fig. 1K; Fig. S10). Furthermore, we compared the survival outcomes between the *CENPM* alteration group and the non-alteration group across pan-cancer cohorts, revealing a negative correlation between *CENPM* alterations and patient survival (Fig. 1L). *CENPM* correlates with TMB, MSI, and MMR dynamics across cancers. Its expression negatively impacted TMB in select tumors, potentially hindering immunotherapy efficacy, while positively correlating with TMB and MSI in others, suggesting enhanced immune recognition (Fig. S11A and S11B). *CENPM* also exhibited tissue-specific correlations with MMR genes (Fig. 1M). These findings suggest *CENPM* as a promising therapy target for immune-cold tumors resistant to conventional immune checkpoint inhibitors.

However, our study has some limitations. While multi-database analysis provides comprehensive insights, experimental validation through cellular and animal models remains essential to confirm *CENPM*’s mechanistic roles. In summary, our research reveals the pan-cancer impact of *CENPM* on tumor microenvironment modulation and its strong association with poor clinical prognosis, suggesting that *CENPM* may function as an independent prognostic biomarker in multiple cancer types and holds potential as a new therapeutic target.

## CRediT authorship contribution statement

**Jinyuan Tang:** Writing – review & editing, Visualization, Investigation, Data curation, Writing – original draft, Software, Formal analysis, Conceptualization. **Sihang Zhang:** Writing – original draft, Software, Data curation, Writing – review & editing, Visualization, Investigation, Conceptualization. **Yongshuai Jiang:** Resources, Conceptualization, Funding acquisition. **Mingming Zhang:** Supervision, Resources, Funding acquisition, Writing – review & editing, Software, Project administration, Conceptualization.

## Data availability

All data generated or analyzed during the current study are available from the corresponding author upon reasonable request.

## Funding

This work was supported by the Program for Young Talents of Basic Research in Universities of Heilongjiang Province, China (No. YQJH2023036).

## Conflict of interests

The authors declare that they have no known competing financial interests or personal relationships that could have appeared to influence the work reported in this paper.
