# Supplementary material for: Comprehensive pan-cancer analysis reveals prognostic significance of CENPM and its role in immune infiltration
Source: Genes Dis. 2025 Aug 16;13(4):101815. doi: 10.1016/j.gendis.2025.101815 (PMC13015223; doi:10.1016/j.gendis.2025.101815)
Supplement: Multimedia component 1 [file mmc1.docx]

**Supplementary material**

**Comprehensive Pan-Cancer Analysis Reveals Prognostic Significance of *CENPM* and Its Role in Immune Infiltration**

Jinyuan Tang^1,†^, Sihang Zhang^1,†^, Yongshuai Jiang^2,*^, Mingming Zhang^2,*^

1 The Second Affiliated Hospital of Harbin Medical University, Harbin Medical University, Harbin 150001, China.

2 College of Bioinformatics Science and Technology, Harbin Medical University, Harbin 150001, China.

† These authors contributed equally to this work.

* Correspondence:

Mingming Zhang, College of Bioinformatics Science and Technology, Harbin Medical University, 194 Xuefu Road, Nangang District, Harbin, Heilongjiang Province, China. E-mail: [zhangmingming@hrbmu.edu.cn](mailto:zhangmingming@hrbmu.edu.cnzhangmingming@hrbmu.edu.cn)

Yongshuai Jiang, College of Bioinformatics Science and Technology, Harbin Medical University, 194 Xuefu Road, Nangang District, Harbin, Heilongjiang Province, China. E-mail: [jiangyongshuai@hrbmu.edu.cn](mailto:jiangyongshuai@hrbmu.edu.cn)

**Figure. S1**


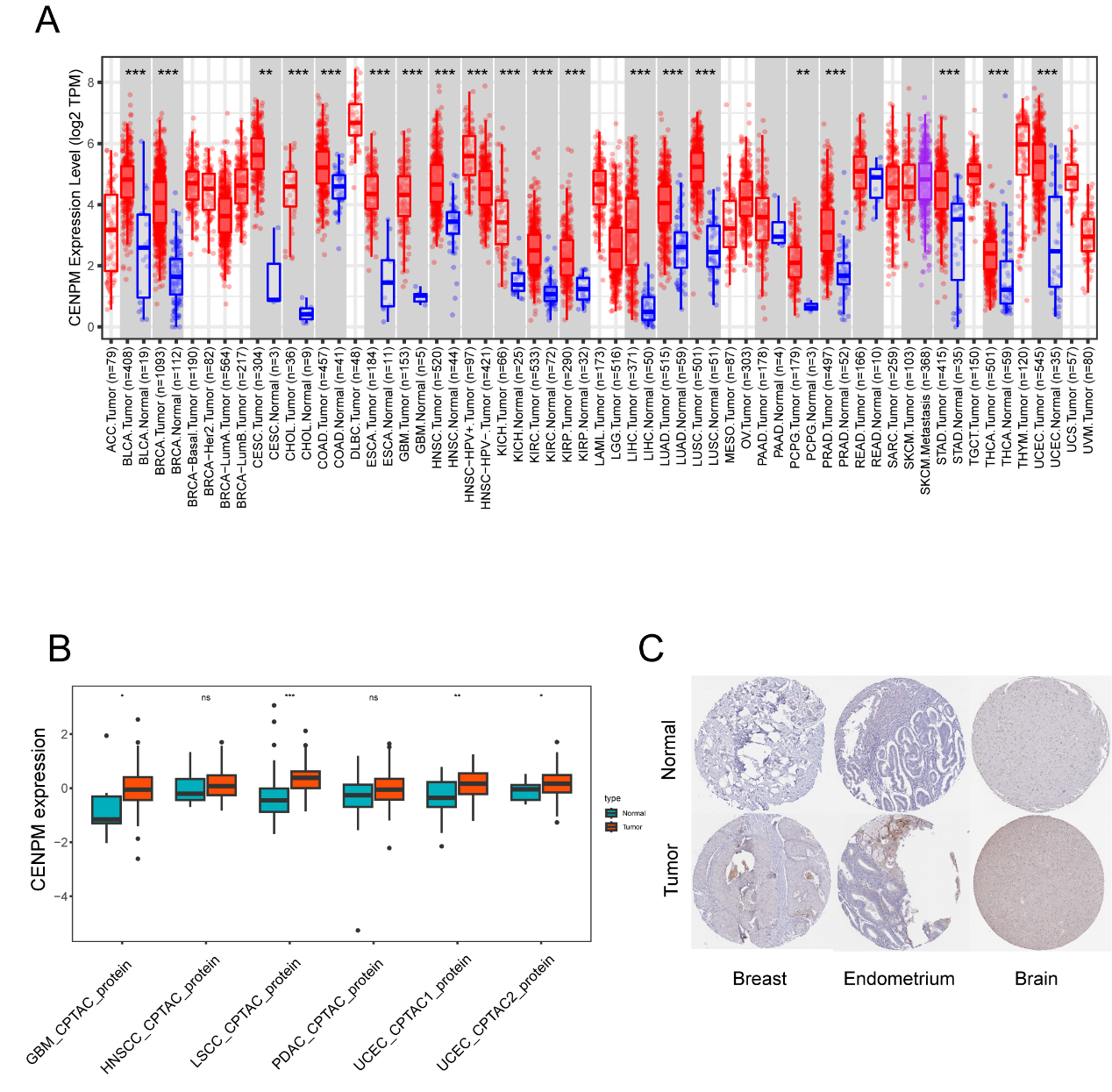


The expression of *CENPM* in normal and tumor tissues. (A) The mRNA expression of *CENPM* in pan-cancers by TIMER2.0 Cancer Exploration; (B)The differential mRNA levels of *CENPM* in six different paired tumors; (C) The immunohistochemical picture about *CENPM* in HPA. (* p < 0.05, ** p < 0.01, *** p < 0.001)

**Figure. S2**


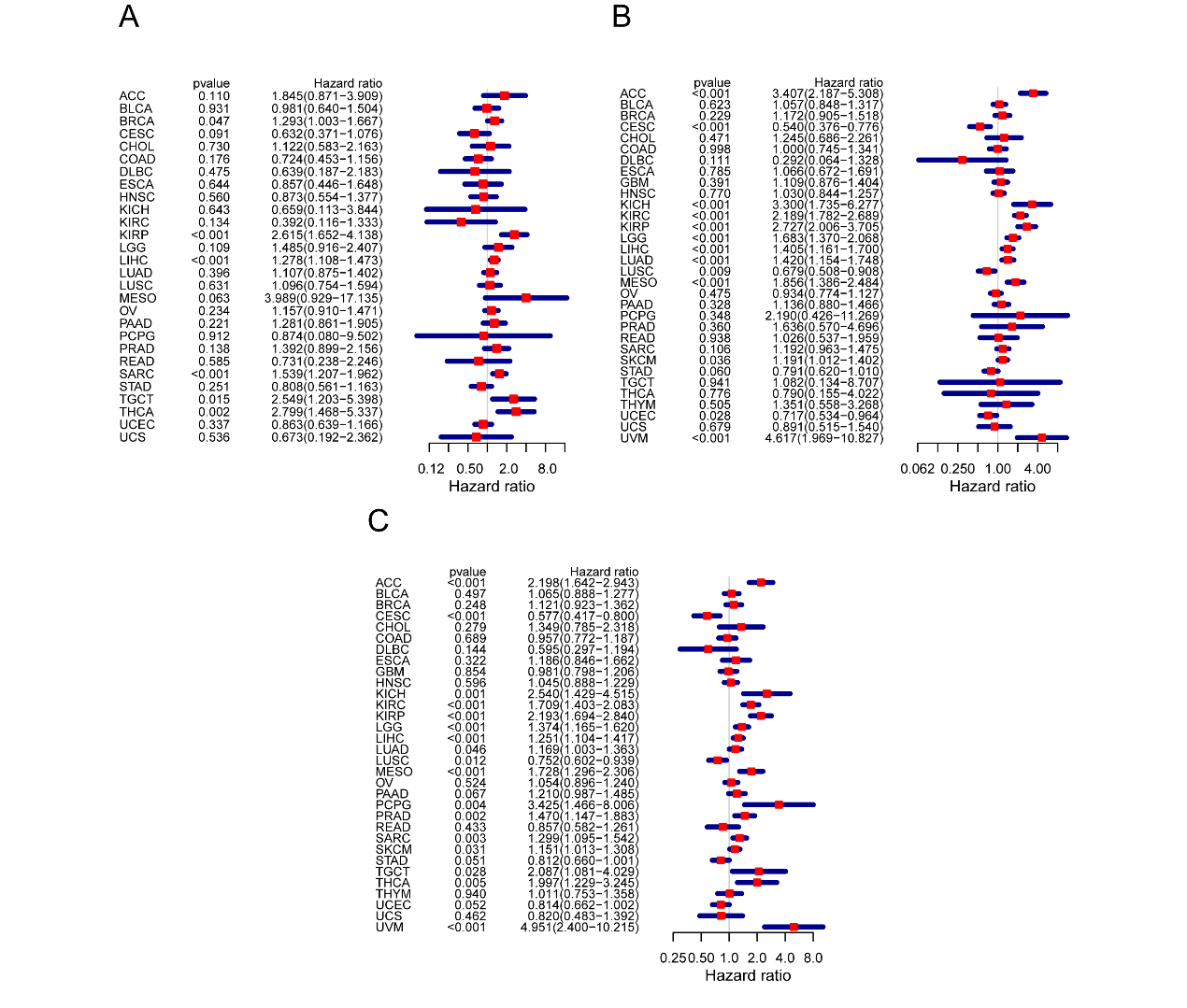


The pan-cancer Cox regression forest plots depicting the association between *CENPM* expression and patient DFI (A), DSS (B), and PFI (C).

**Figure. S3**


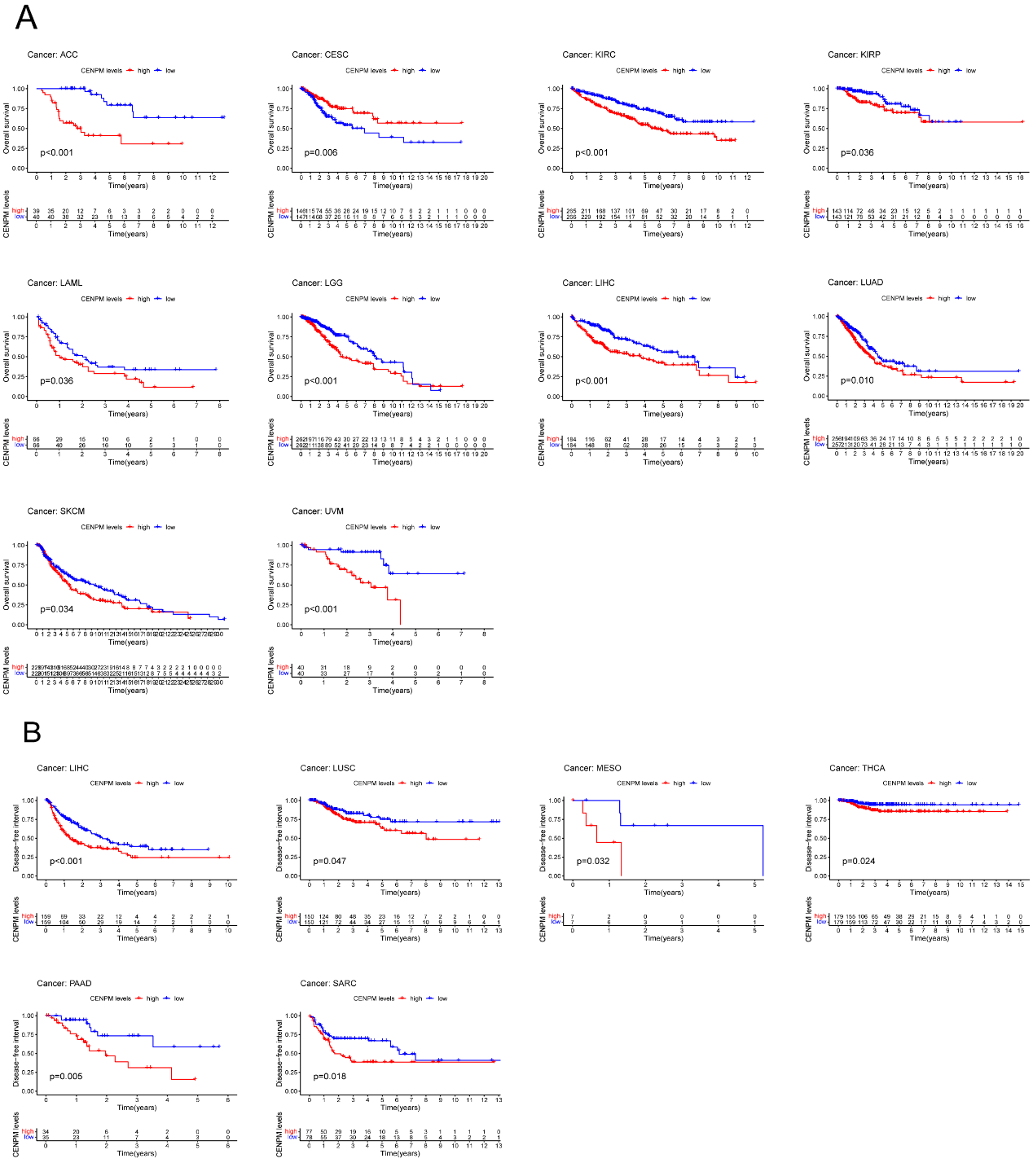


Kaplan–Meier analysis of OS (A) and DFI (B) comparing high and low expression of *CENPM* in various types of tumors.

**Figure. S4**


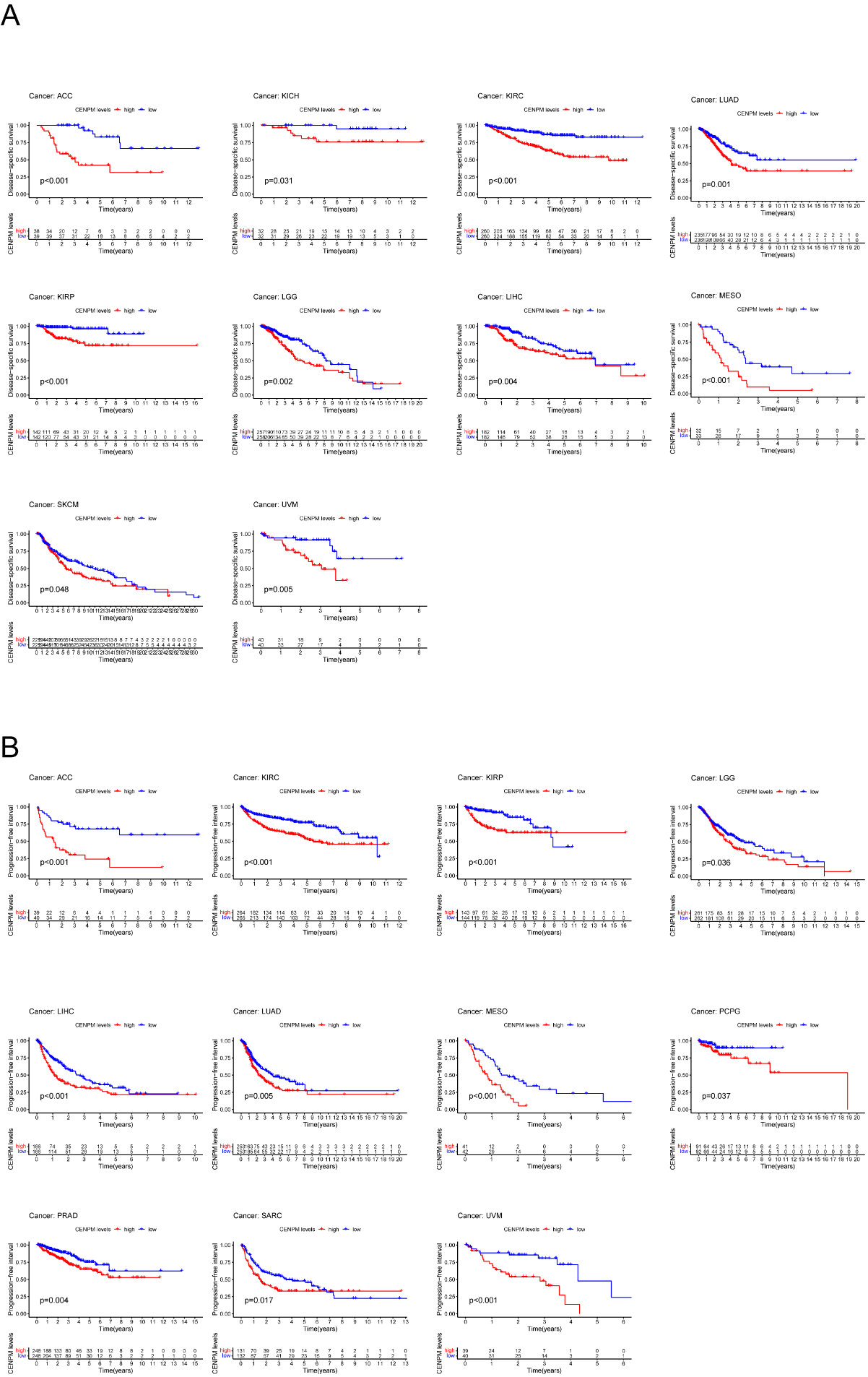


Kaplan–Meier analysis of DSS (A) and PFI (B) comparing high and low expression of *CENPM* in various types of tumors.

**Figure. S5**


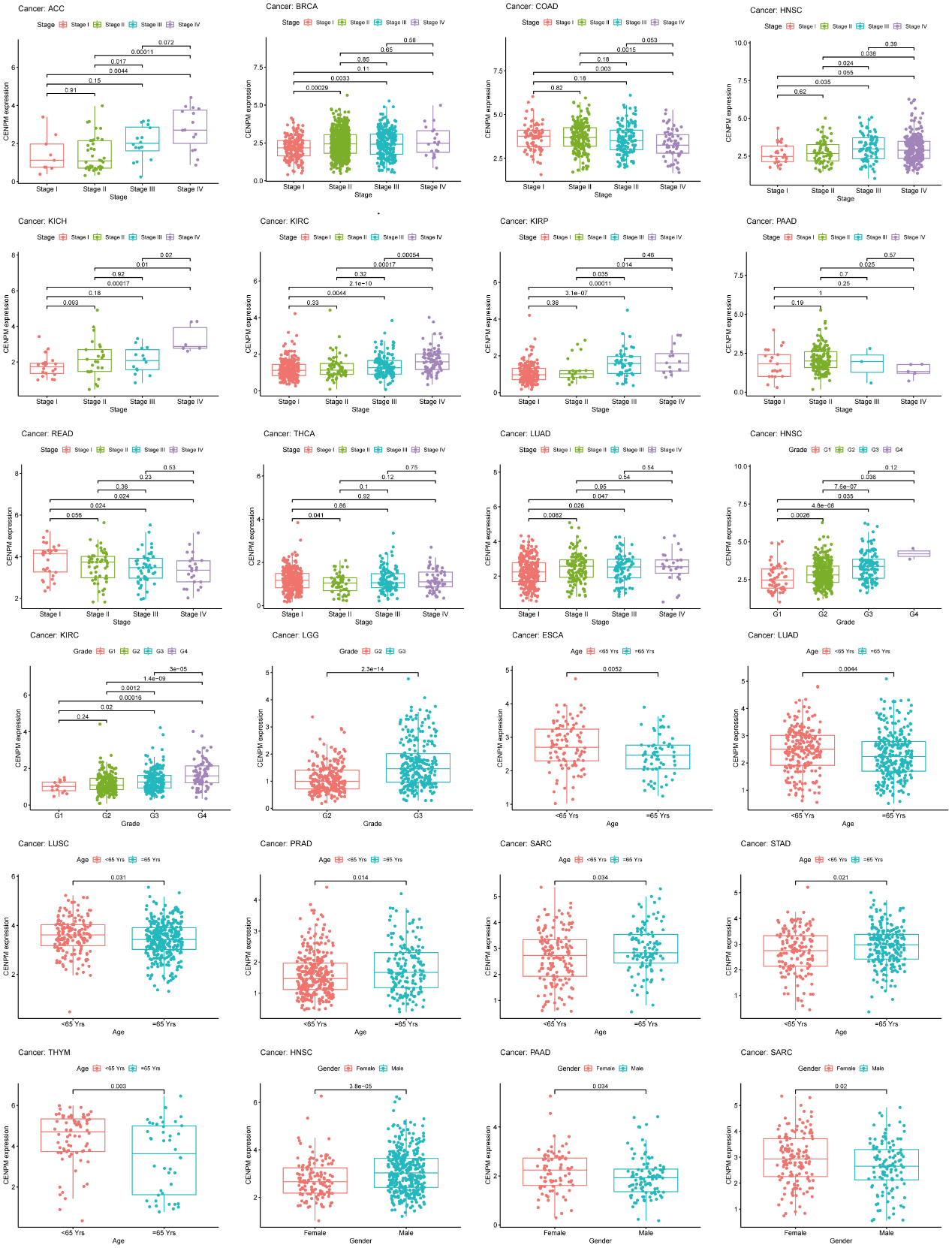


*CENPM* expression is correlated with various clinical features (stage, grade, age and gender) in pan-cancer.

**Figure. S6**


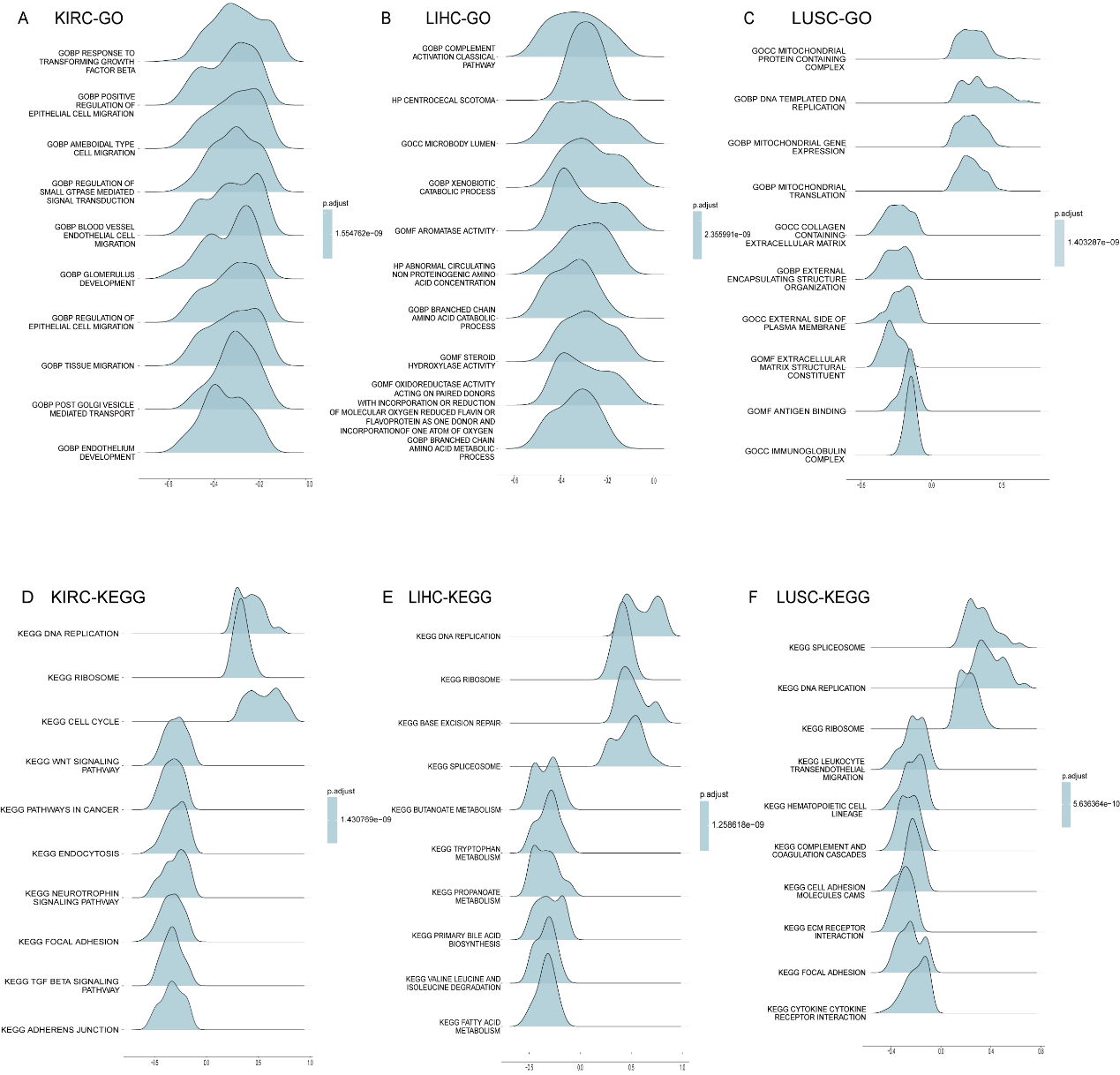


GSEA analysis of *CENPM* in pan-cancer. (A-C) GSEA-GO functional enrichment analysis results in KIRC (A), LIHC (B), and LUSC (C). (D-F) GSEA-KEGG functional enrichment analysis results in KIRC (D), LIHC (E), and LUSC (F).

**Figure. S7**


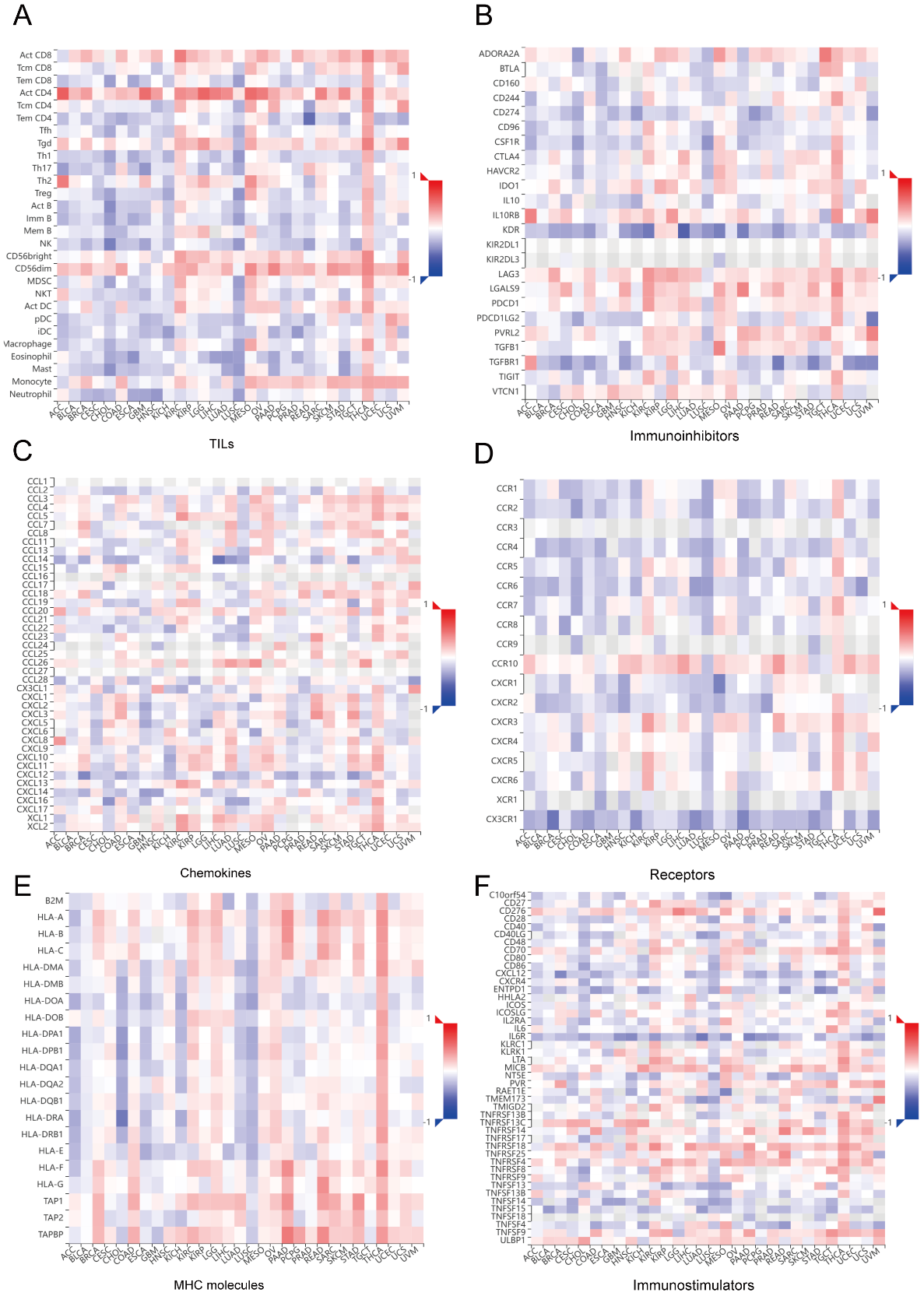


The relationship between *CENPM* expression and tumor-infiltrating lymphocytes (TILs) as well as tumor-related genes from TISIDB database. (A-F) The correlation between *CENPM* expression and TILs (A), immunoinhibitors (B), chemokines (C), chemokine receptors (D), MHC molecules (E), immunostimulators (F) in pan-cancer.

**Figure. S8**


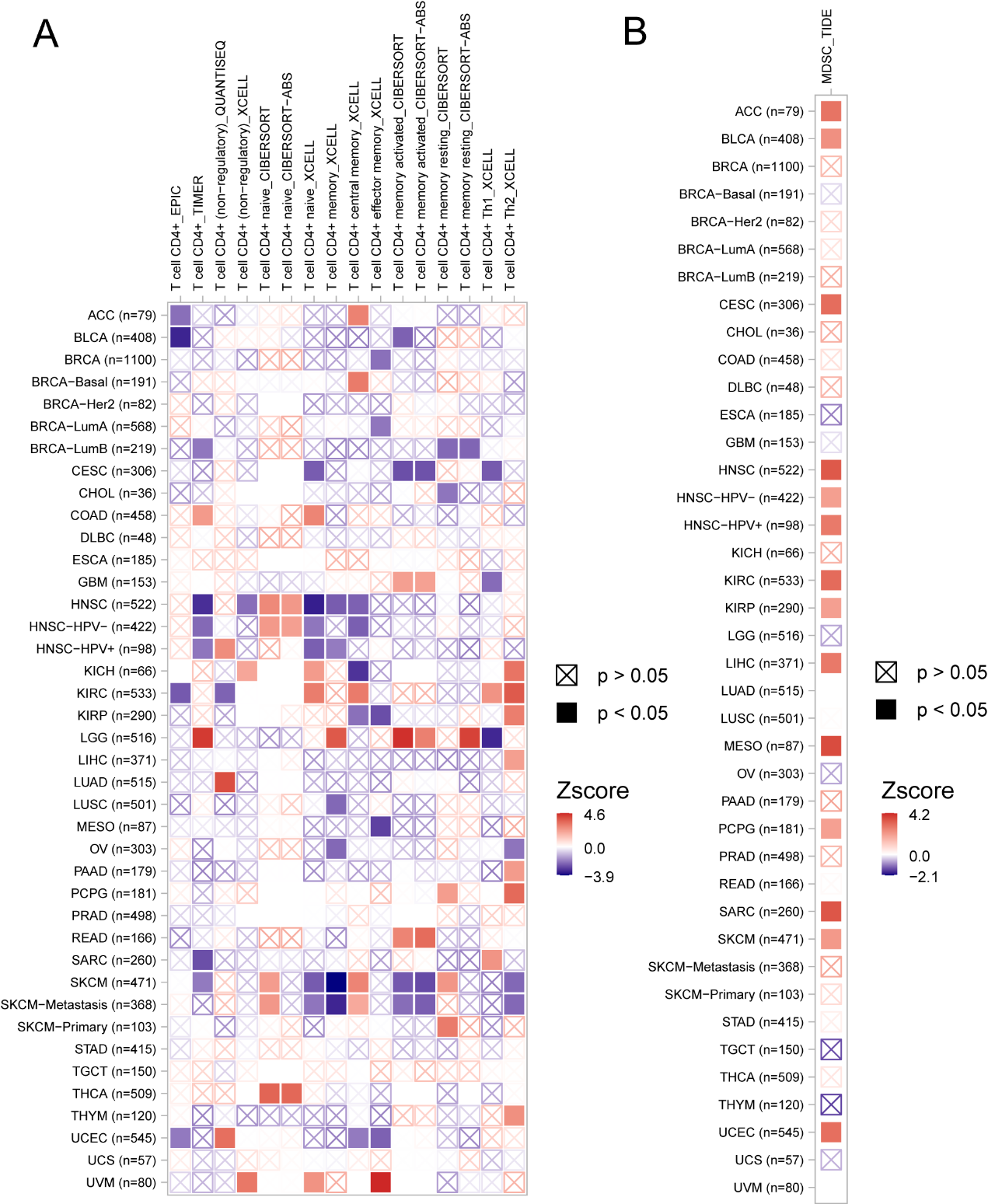


The correlation between immune infiltration and patient prognosis. (A, B) The correlation between various CD4+T cell infiltrations in pan-cancer and patient prognosis (A). The correlation between MDSC cell infiltration in pan-cancer and patient prognosis (B).

**Figure. S9**


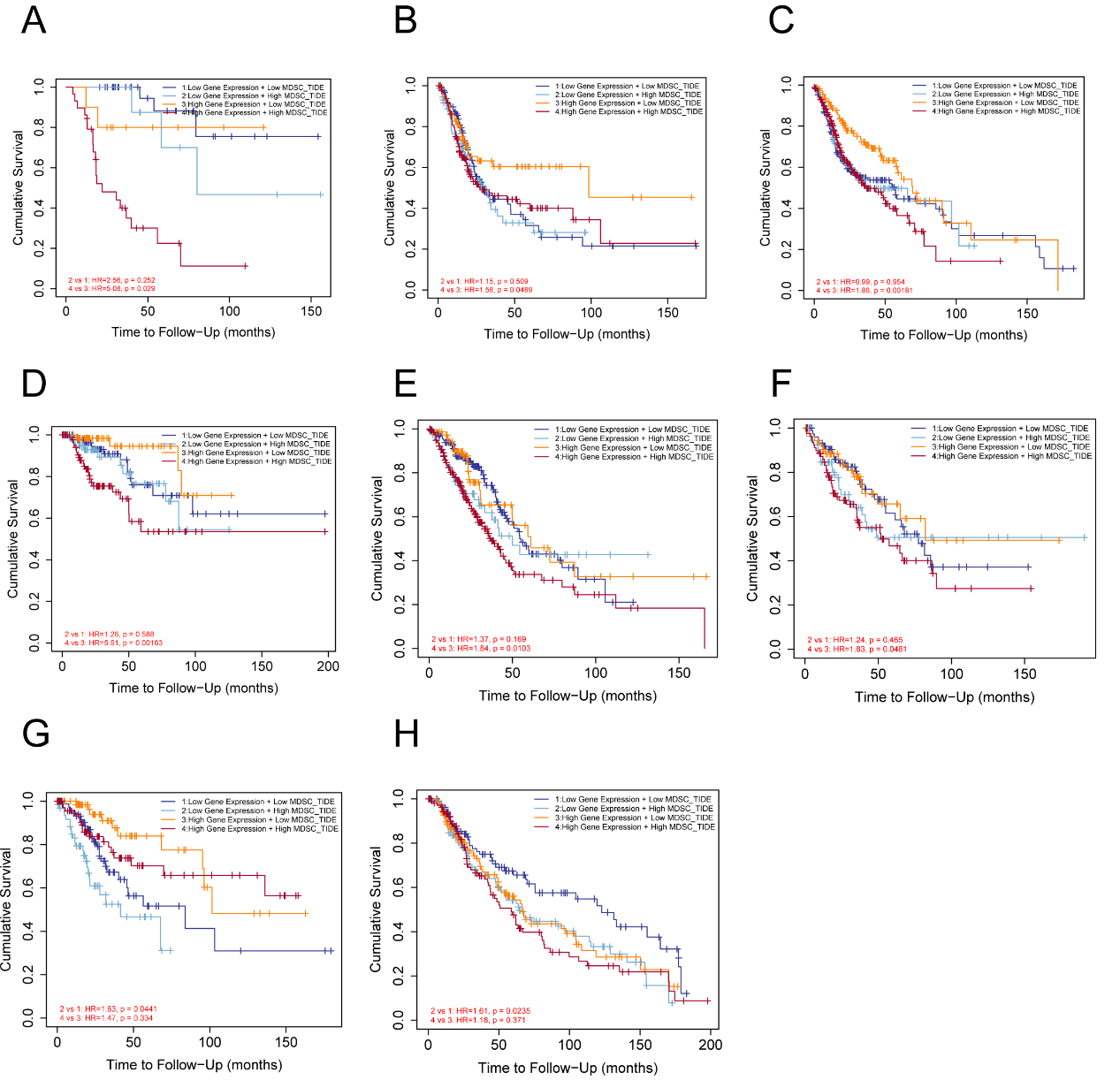


Effects of *CENPM* expression and MDSC cell infiltration on the prognosis of patients with ACC (A), BLCA (B), HNSC (C), KIRP (D), LUAD (E), SARC (F), MESO (G), and SKCM (H).

**Figure. S10**


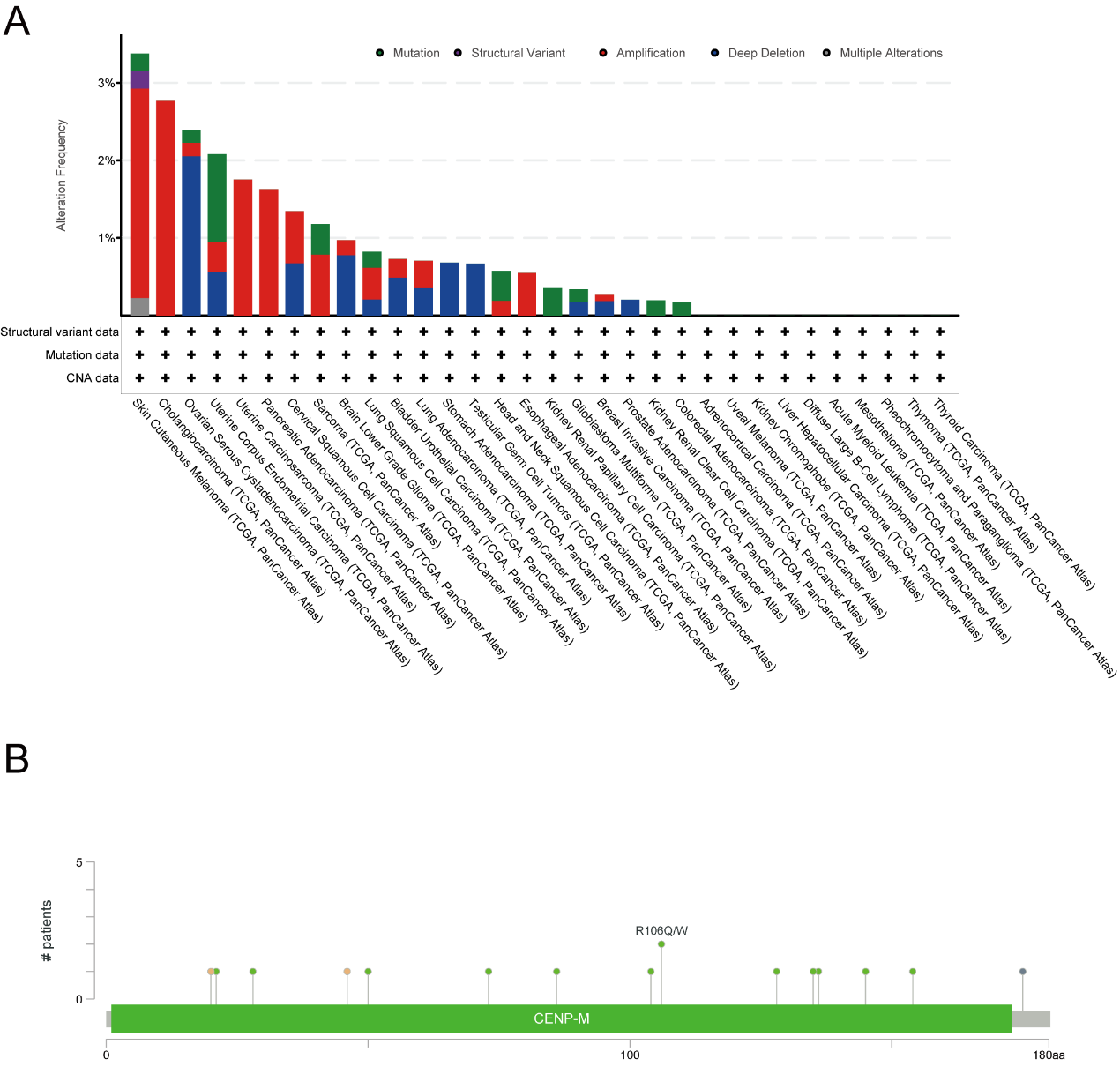


Mutation status of *CENPM* in TCGA tumors. (A, B) Mutation status of *CENPM* in TCGA tumors was analyzed using the cBioPortal tool. The alteration frequency with mutation types (A) and mutation site (B) are displayed.

**Figure. S11**


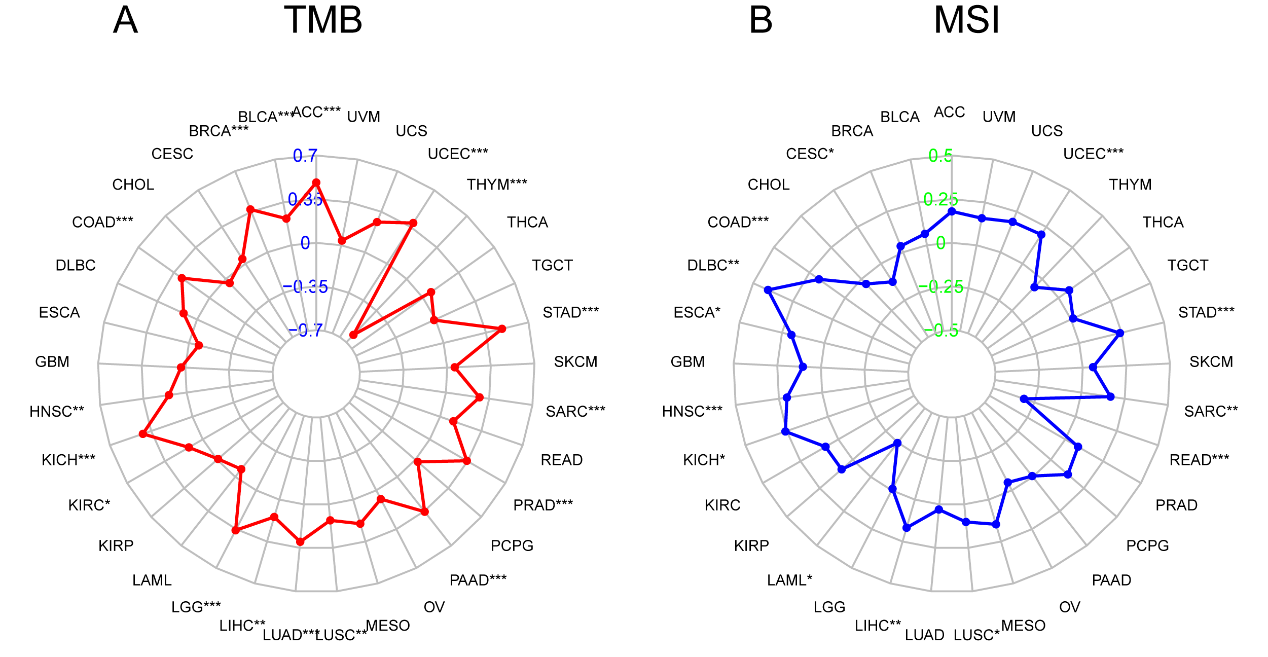


Relationship between *CENPM* gene expression and genetic alteration in pan-cancer. (A, B) The radar charts display the correlations between *CENPM* expression and TMB (A) and MSI (B) in cancers. The gray circles indicate correlation coefficients, the number of which were shown on the graphs. (* p < 0.05, ** p < 0.01, *** p < 0.001)

**Table S1**

Results of GSEA analysis based on GO and KEGG in Pan-Cancer

| Analysis | Biological processes | Frequency |
| --- | --- | --- |
| GSEA-GO | GOCC_MITOCHONDRIAL_PROTEIN_CONTAINING_COMPLEX | 0.64 |
|  | GOCC_ORGANELLAR_RIBOSOME | 0.64 |
|  | GOCC_RIBOSOMAL_SUBUNIT | 0.61 |
|  | GOMF_STRUCTURAL_CONSTITUENT_OF_RIBOSOME | 0.58 |
|  | GOCC_INNER_MITOCHONDRIAL_MEMBRANE_PROTEIN_COMPLEX | 0.52 |
|  | GOBP_MITOCHONDRIAL_TRANSLATION | 0.48 |
|  | GOCC_LARGE_RIBOSOMAL_SUBUNIT | 0.48 |
|  | GOBP_OXIDATIVE_PHOSPHORYLATION | 0.42 |
|  | GOCC_RIBOSOME | 0.39 |
|  | GOBP_ATP_SYNTHESIS_COUPLED_ELECTRON_TRANSPORT | 0.39 |
| GSEA-KEGG | KEGG_DNA_REPLICATION | 0.94 |
|  | KEGG_CELL_CYCLE | 0.85 |
|  | KEGG_RIBOSOME | 0.76 |
|  | KEGG_HUNTINGTONS_DISEASE | 0.73 |
|  | KEGG_PROTEASOME | 0.70 |
|  | KEGG_PARKINSONS_DISEASE | 0.70 |
|  | KEGG_OXIDATIVE_PHOSPHORYLATION | 0.70 |
|  | KEGG_SPLICEOSOME | 0.61 |
|  | KEGG_PYRIMIDINE_METABOLISM | 0.55 |
|  | KEGG_ALZHEIMERS_DISEASE | 0.42 |

**Table S2**

Correlations between *CENPM* expression in 33 tumor types and Stromal scores, Immune scores, and ESTIMATE scores.

|  | Stromal scores | | Immune scores | | ESTIMATE scores | |
| --- | --- | --- | --- | --- | --- | --- |
|  | spearman_R | spearman_P | spearman_R | spearman_P | spearman_R | spearman_P |
| ACC | -0.247311828 | 0.030123481 | -0.238977837 | 0.036335361 | -0.235126324 | 0.039548625 |
| BLCA | -0.09924427 | 0.045932903 | -0.068710774 | 0.167544989 | -0.090883337 | 0.067681485 |
| BRCA | -0.379638413 | 2.98E-38 | -0.020840257 | 0.4944749 | -0.194899429 | 1.11E-10 |
| CESC | -0.24627716 | 2.15E-05 | -0.178085422 | 0.002294171 | -0.234978342 | 5.17E-05 |
| CHOL | -0.234764142 | 0.168134213 | -0.259990991 | 0.125679541 | -0.273634083 | 0.106342578 |
| COAD | -0.247810113 | 2.57E-05 | -0.005261427 | 0.929906448 | -0.135410096 | 0.022947539 |
| DLBC | -0.117360469 | 0.437299404 | -0.410422448 | 0.004611195 | -0.322849214 | 0.028641824 |
| ESCA | -0.175605965 | 0.018050855 | -0.273146284 | 0.000198949 | -0.241491594 | 0.001056478 |
| GBM | -0.269069076 | 0.00080242 | -0.266376318 | 0.000909439 | -0.279452256 | 0.000489192 |
| HNSC | -0.164141968 | 0.000177809 | 0.042909474 | 0.330182232 | -0.049554127 | 0.260713041 |
| KICH | -0.1625 | 0.195903209 | -0.12145979 | 0.335139111 | -0.15472028 | 0.218465961 |
| KIRC | -0.04206019 | 0.334742221 | 0.2348503 | 4.76E-08 | 0.146623409 | 0.000726321 |
| KIRP | 0.048044737 | 0.41909598 | 0.040746868 | 0.493250687 | 0.055933309 | 0.34678183 |
| LAML | 0.018946213 | 0.818603972 | 0.001665163 | 0.983919939 | 0.022486951 | 0.785459819 |
| LGG | 0.008090484 | 0.856224016 | 0.098946322 | 0.026332196 | 0.068515756 | 0.124498836 |
| LIHC | -0.251916912 | 1.16E-06 | 0.06127569 | 0.244210632 | -0.075926581 | 0.14882591 |
| LUAD | -0.171487299 | 0.000116414 | -0.104657665 | 0.01924295 | -0.149256851 | 0.000814255 |
| LUSC | -0.41408596 | 9.17E-22 | -0.358275412 | 2.57E-16 | -0.405131393 | 8.09E-21 |
| MESO | 0.232501783 | 0.03225317 | 0.152952442 | 0.162260085 | 0.187289303 | 0.086092705 |
| OV | -0.067103701 | 0.171915663 | 0.09030168 | 0.065765454 | 0.016905504 | 0.731001932 |
| PAAD | -0.203813572 | 0.006509022 | -0.10197225 | 0.17683525 | -0.150456679 | 0.045618426 |
| PCPG | -0.16923449 | 0.024332778 | -0.128474298 | 0.088343958 | -0.153191839 | 0.041782872 |
| PRAD | 0.033795625 | 0.453123094 | 0.112309719 | 0.012407723 | 0.089892555 | 0.04561103 |
| READ | -0.191861761 | 0.068468751 | -0.107055264 | 0.312480519 | -0.168561873 | 0.110220998 |
| SARC | -0.23707968 | 0.000120786 | 0.009121983 | 0.88406956 | -0.096063299 | 0.12378623 |
| SKCM | -0.071580425 | 0.128622479 | 0.031651282 | 0.502080154 | -0.008355633 | 0.859387113 |
| STAD | -0.34234195 | 4.14E-12 | -0.126821122 | 0.012414809 | -0.259784036 | 2.10E-07 |
| TGCT | -0.233702841 | 0.006997642 | -0.049271725 | 0.574766337 | -0.143119151 | 0.101606716 |
| THCA | 0.217830972 | 8.11E-07 | 0.321502062 | 1.47E-13 | 0.292894625 | 2.08E-11 |
| THYM | -0.291605038 | 0.001356028 | 0.331456181 | 0.000245975 | 0.08634536 | 0.352524458 |
| UCEC | -0.113072491 | 0.132898226 | 0.028244717 | 0.70821734 | -0.037132631 | 0.622655328 |
| UCS | 0.012474581 | 0.92729455 | 0.09073976 | 0.505990949 | 0.066747552 | 0.625002507 |
| UVM | 0.174553908 | 0.123907359 | 0.242471823 | 0.031316646 | 0.230811364 | 0.040705322 |
