# Supplementary material for: Comprehensive pan-cancer analysis reveals prognostic significance of CENPM and its role in immune infiltration
Source: Genes Dis. 2025 Aug 16;13(4):101815. doi: 10.1016/j.gendis.2025.101815 (PMC13015223; doi:10.1016/j.gendis.2025.101815)
Supplement: Multimedia component 2 [file mmc2.docx]

**Materials and Methods**

**Comprehensive Pan-Cancer Analysis Reveals Prognostic Significance of *CENPM* and Its Role in Immune Infiltration**

Jinyuan Tang^1,†^, Sihang Zhang^1,†^, Yongshuai Jiang^2,*^, Mingming Zhang^2,*^

1 The Second Affiliated Hospital of Harbin Medical University, Harbin Medical University, Harbin 150001, China.

2 College of Bioinformatics Science and Technology, Harbin Medical University, Harbin 150001, China.

† These authors contributed equally to this work.

* Correspondence:

Mingming Zhang, College of Bioinformatics Science and Technology, Harbin Medical University, 194 Xuefu Road, Nangang District, Harbin, Heilongjiang Province, China. E-mail: [zhangmingming@hrbmu.edu.cn](mailto:zhangmingming@hrbmu.edu.cnzhangmingming@hrbmu.edu.cn)

Yongshuai Jiang, College of Bioinformatics Science and Technology, Harbin Medical University, 194 Xuefu Road, Nangang District, Harbin, Heilongjiang Province, China. E-mail: [jiangyongshuai@hrbmu.edu.cn](mailto:jiangyongshuai@hrbmu.edu.cn)

# Materials and Methods

## *Data Collection*

RNA sequencing data (HTSeq-FPKM), mutation data, survival data, and clinically pertinent pathological characteristics for 33 cancer types (Table 1) were sourced from TCGA. These datasets were retrieved from the University of California, Santa Cruz (UCSC) Xena platform^1^ (<https://xena.ucsc.edu/>). Additionally, normal tissue expression data were acquired from the GTEx project (<https://www.gtexportal.org/>). The CPTAC (<https://pdc.cancer.gov/pdc/>) serves as a repository for proteomic and genomic data across various tumor types. The proteomic data utilized for the analysis of *CENPM*-expressing tumors was obtained from this consortium^2^. Furthermore, immunohistochemical images of *CENPM* (antibody HPA042404) in various tumor tissues and their corresponding normal tissues were retrieved from the HPA database^3^ (<https://www.proteinatlas.org/>).

## *CENPM Expression Analysis*

To investigate the gene expression profile of *CENPM*, the "Gene DE" module of the Tumor Immune Estimation Resource 2.0 (TIMER2.0; <http://timer.comp-genomics.org/>) database was employed to assess differential gene expression between normal and tumor tissues across various cancer types, and the Wilcoxon test was used to calculate statistical significance^4^. Due to the absence of normal samples in the TCGA database, the Sangerbox platform (<http://vip.sangerbox.com/>) was utilized to analyze the expression difference of *CENPM* between normal and tumor tissues within the standardized pan-cancer dataset TCGA TARGET GTEx, which was obtained from UCSC Xena^5^. Unpaired Wilcoxon Rank-Sum test and Signed-Rank test were employed for significance analysis. The "expression DIY" module of the Gene Expression Profiling Interactive Analysis 2 (GEPIA2) online tool was utilized to evaluate the differential expression of *CENPM* and to corroborate the previously acquired findings^6^. A p-value cutoff of 0.01 and a log2FC cutoff of 1 were used with "Match TCGA and GTEx data" was set. The log2(TPM + 1) data was adopted for log-scale. Additionally, the R package "PCAs" was employed to analyze proteomic data from CPTAC^7^.

## *Survival Analysis and ROC Analysis*

Kaplan-Meier curves were generated to evaluate patient outcomes, including overall survival (OS), disease-free interval (DFI), disease-specific survival (DSS), and progression-free interval (PFI). Here, tumors were categorized into high and low expression groups based on the median expression level of *CENPM*. Additionally, the "survival" package was utilized to construct a univariate Cox proportional hazards regression model, with the findings represented in forest plots. Furthermore, the "survival analysis" feature of the GEPIA2 platform was utilized to create survival maps for OS across 33 different tumor types. Time-dependent receiver operating characteristic (ROC) curves were generated using the "timeROC" package to assess the predictive accuracy of *CENPM* regarding patient prognosis. The R packages "survival" and "survminer" were used to assess the influence of *CENPM* on the prognosis of patients with various cancers.

## *Clinical Correlation Analysis*

The "limma" and "ggpubr" packages were employed to investigate the correlation between *CENPM* expression and various clinicopathological characteristics, including age, gender, stage, and grade, across pan-cancer datasets. Boxplots were utilized to illustrate the correlation between *CENPM* expression and the distinct clinicopathological features.

## *Enrichment Analysis*

To investigate the functional pathways and effects associated with *CENPM*, the Gene Ontology (GO) gene set and the Kyoto Encyclopedia of Genes and Genomes (KEGG) gene set were obtained from the MSigDB database^8^ (<https://www.gsea-msigdb.org/gsea/msigdb>). Subsequently, the Spearman correlation coefficient was computed between *CENPM* and all genes within the TCGA pan-cancer dataset. The genes that exhibited significant correlations (P < 0.05) were then ranked in descending order. Following this, GSEA function from the "clusterProfiler" package was employed to conduct a functional enrichment analysis.

## *Mutation Profiles Analysis*

The cBioPortal database (<https://www.cbioportal.org/>) was utilized to investigate the mutation status of *CENPM* within the TCGA Pan-Cancer dataset and its implications for survival outcomes.^9^. The "Cancer Types Summary" module of cBioPortal provides an overview of the mutation frequency of *CENPM* across various cancer types. The "Mutations" and "Plots" modules were employed to pinpoint specific mutation sites and to quantify mutation counts, respectively. In addition, we assessed the OS differences between groups with alterations in *CENPM* and those without such changes using the "Comparison/Survival" module.

## *Analysis of MMR, TMB, and MSI in Cancers*

MMR is a critical mechanism responsible for rectifying base mismatches that occur during DNA replication, thereby contributing to genomic stability^10^. Dysregulation of MMR can lead to tumorigenesis. The Pearson correlations coefficient between *CENPM* and the expression levels of five MMR genes (*MLH1*, *MSH2*, *MSH6*, *PMS2*, and *EPCAM*) were computed based on expression profile data from TCGA database. TMB is defined as the count of somatic nonsynonymous mutations within a specific genomic region and has been associated with the effectiveness of immune checkpoint inhibitors (ICIs) ^11^. Additionally, MSI, which is characterized by the insertion or deletion of nucleotides in microsatellite regions, also influences patient responses to immunotherapy^12^. The correlation between *CENPM* expression and TMB/MSI within the TCGA pan-cancer mutation dataset was examined using the R language, with statistical significance determined through the Spearman correlation test.

## *TME and Immune Infiltration*

The immune score, immune cell infiltration and immune-related genes in pan-cancer were analyzed to research the correlation between expression of *CENPM* and TME. The R package "ESTIMATE" (version 1.0.13) was employed to compute the immune score, stromal score, and ESTIMATE score for 33 distinct tumor types. The Spearman's correlation coefficient between *CENPM* and immune infiltration scores in various tumors was calculated using the "corr.test" function from the R package psych (version 2.1.6). A high immune score and stromal score signify substantial presence of immune and stromal components within the tumor tissue, whereas the ESTIMATE score is indicative of tumor purity. The TIMER2.0 database facilitated the assessment of the correlation between the infiltration of various immune cells and *CENPM* expression across pan-cancer. Additionally, the TISIDB web portal (<http://cis.hku.hk/TISIDB/>) was utilized to explore the relationship between *CENPM* and tumor-infiltrating lymphocytes (TILs), as well as immunosuppressants, immunostimulants, chemokines, chemokine receptors, and the correlations involving MHC molecules.

## *Statistical Analysis*

Use Perl scripts to organize and normalize the data matrix. For the comparison of two groups, Student's t-test was utilized, while the Kruskal-Wallis test or one-way analysis of variance was applied for the comparison of multiple groups. Correlation analyses between two variables were conducted using either the Spearman or Pearson correlation coefficients. Statistical analyses were performed using R software version 4.4.1, with significance threshold set at p < 0.05.

# Reference:

1. Goldman MJ, Craft B, Hastie M, et al. Visualizing and interpreting cancer genomics data via the Xena platform. Nat Biotechnol. 2020;38(6):675-678.

2. Edwards NJ, Oberti M, Thangudu RR, et al. The CPTAC Data Portal: A Resource for Cancer Proteomics Research. J Proteome Res. 2015;14(6):2707-2713.

3. Interactive human protein atlas launches. Cancer Discov. 2015;5(4):339.

4. Li T, Fu J, Zeng Z, et al. TIMER2.0 for analysis of tumor-infiltrating immune cells. Nucleic Acids Res. 2020;48(W1):W509-w514.

5. Shen W, Song Z, Zhong X, et al. Sangerbox: A comprehensive, interaction-friendly clinical bioinformatics analysis platform. iMeta. 2022;1(3):e36.

6. Tang Z, Kang B, Li C, Chen T, Zhang Z. GEPIA2: an enhanced web server for large-scale expression profiling and interactive analysis. Nucleic Acids Res. 2019;47(W1):W556-w560.

7. Wang J, Song X, Wei M, et al. PCAS: An Integrated Tool for Multi-Dimensional Cancer Research Utilizing Clinical Proteomic Tumor Analysis Consortium Data. Int J Mol Sci. 2024;25(12).

8. Liberzon A, Birger C, Thorvaldsdóttir H, Ghandi M, Mesirov JP, Tamayo P. The Molecular Signatures Database (MSigDB) hallmark gene set collection. Cell Syst. 2015;1(6):417-425.

9. Cerami E, Gao J, Dogrusoz U, et al. The cBio cancer genomics portal: an open platform for exploring multidimensional cancer genomics data. Cancer Discov. 2012;2(5):401-404.

10. He Y, Zhang L, Zhou R, Wang Y, Chen H. The role of DNA mismatch repair in immunotherapy of human cancer. Int J Biol Sci. 2022;18(7):2821-2832.

11. Qian ZY, Pan YQ, Li XX, et al. Modulator of TMB-associated immune infiltration (MOTIF) predicts immunotherapy response and guides combination therapy. Sci Bull (Beijing). 2024;69(6):803-822.

12. Wilbur HC, Le DT, Agarwal P. Immunotherapy of MSI Cancer: Facts and Hopes. Clin Cancer Res. 2024;30(8):1438-1447.
